# Usefulness of a new semiological classification for characterizing psychogenic nonepileptic seizures

**DOI:** 10.1590/0004-282X-ANP-2019-0171

**Published:** 2021-05-07

**Authors:** Bárbara Ingrid ROSSO, Juan Carlos AVALOS, Ana Gabriela BESOCKE, Maria del Carmen GARCÍA

**Affiliations:** 1 Hospital Italiano de Buenos Aires, Buenos Aires, Argentina. Hospital Italiano de Buenos Aires Buenos Aires Argentina

**Keywords:** Stress, Psychological, Seizures, Diagnosis, Estrés Psicológico, Convulsiones, Diagnóstico

## Abstract

**Background:**

Nonepileptic events misdiagnosed as epilepsy lead to a risk of iatrogenic morbidity, which increases health costs. Among the patients affected by nonepileptic events, 11-46% are psychogenic nonepileptic seizures (PNESs).

**Objective:**

To investigate the usefulness of the semiological classification of PNESs among patients diagnosed by means of video electroencephalograms (vEEGs).

**Methods:**

This was a retrospective review of the medical records of patients admitted to the adult vEEG unit between April 2007 and December 2016, who were diagnosed with PNES that was confirmed through vEEG. Analysis on demographic and clinical data and classification of PNESs according to the Magaudda classification were performed.

**Results:**

We identified 143 patients, among whom 31.5% had also epilepsy. According to the Magaudda classification, the events were: hypermotor (58%); subjective symptoms (21.7%); akinetic (14.7%) and focal motor (5.6%). Hypermotor predominated in both genders, followed by subjective symptoms in women (23.9%) and akinetic in men (19.2%). The mean number of antiepileptic drugs (AEDs) prescribed per patient was 2.3. Thirty-two patients (22.4%) required at least one hospitalization for PNESs. 48.3% of the patients had psychiatric comorbidities.

**Conclusion:**

The proposed semiological classification of PNESs is a relevant tool that general neurologists can use to characterize these events in their daily practice. Correct use of this classification, together with vEEG and appropriate clinical suspicion, makes it possible to reach an accurate early diagnosis, thus reducing morbidity and, possibly, the high costs associated with PNESs

## RESUMEN

**Introducción:**

Los eventos no epilépticos diagnosticados erróneamente como epilepsia conducen a un riesgo de morbilidad iatrogénica que aumenta los costes en salud. Entre los pacientes afectados por eventos no epilépticos, un 11-46% son de origen psicógeno (PNES).

**Objetivos:**

Evaluar la utilidad de la clasificación semiológica de PNES en pacientes diagnosticados por video electroencefalograma (vEEG).

**Métodos:**

Revisión retrospectiva de los registros médicos de pacientes ingresados en la unidad de adultos de vEEG entre 04-2007 y 12-2016, que fueron diagnosticados con PNES confirmado por vEEG. Se realizó un análisis de los datos demográficos y clínicos, y la clasificación de los PNES según la clasificación de Magaudda.

**Resultados:**

Identificamos 143 pacientes, el 31,5% de los cuales también tenía epilepsia. Según la clasificación de Magaudda, los eventos fueron: hipermotor 58%; síntomas subjetivos 21,7%; akinética 14,7% y motor focal 5,6%. El hipermotor predominó en ambos los sexos, seguido de síntomas subjetivos en las mujeres (23,9%) y akinéticos en los hombres (19,2%). La cantidad media de fármacos antiepilépticos (FAE) recetados por paciente fue 2.3. Un total de 32 pacientes (22.4%) requirieron al menos una hospitalización por PNES. El 48,3% de los pacientes tenía comorbilidad psiquiátrica.

**Conclusión:**

La clasificación semiológica de los PNES propuesta es una herramienta relevante que los neurólogos generales pueden usar para caracterizar esos eventos en su práctica diaria. El uso correcto de esta clasificación, vEEG y una sospecha clínica adecuada permite llegar a un diagnóstico preciso y temprano, reduciendo así la morbilidad y, posiblemente, los altos costes asociados con las PNES.

## INTRODUCTION

Psychogenic nonepileptic seizures (PNESs) are characterized by abnormal movements and epileptic seizure-like feelings or experiences, but they are caused by psychological processes. Most of them are classified as conversion disorders^1^ by the DSM-5.

Their annual incidence has been estimated as approximately 1.5 cases per 100,000 individuals. The prevalence of PNESs is very difficult to gauge due to the lack of formal epidemiological studies. Nonetheless, its prevalence is considered to range from 5 to 20% in the adult population with epilepsy and 15 to 40% among adults who have been referred to centers specializing in epilepsy. In the case of children referred to specialist centers for presumed epilepsy, the prevalence of PNESs is 1-9%. Among the patients with nonepileptic seizures, 11-46% of the cases are of psychogenic origin^2^.

Even though several classifications have been proposed, Magaudda et al. have recently validated one that divides PNESs into four groups according to their semiology: hypermotor (H), characterized by generalized tonic, clonic or dystonic movements; akinetic (A), defined by the absence of movement and the possibility of slight shaking; focal motor (FM), presenting focal motor movements; and, lastly, subjective symptoms (SS), which are experiential and/or subjective symptoms reported by the patient^2^.

Our main hypothesis here is that this is a helpful tool for identifying PNES in the clinical setting and that it can contribute to semiological characterization of PNES.

Thus, the objective of this study was to investigate the usefulness of the semiological classification of PNESs proposed by Magaudda et al.^2^ among patients diagnosed by means of video electroencephalograms (vEEGs).

## METHODS

This study, as well as the informed consent statement that was given out to patients and/or relatives, was approved by the Ethics Committee for Research Protocols of the Hospital Italiano de Buenos Aires (protocol no. 4021).

Patients admitted to the adult vEEG unit of the Hospital Italiano de Buenos Aires between 2005 and 2017 were evaluated retrospectively. This study included patients with a diagnosis of PNESs confirmed by means of vEEG, either with or without associated epilepsy.

The analysis material comprised demographic data and the results from interictal electroencephalograms (EEG) that were performed during the natural course of the patient’s disease, even including those done in other centers. The equipment used to obtain vEEG information was the 64-channel Stellate Harmonie. The electrodes were placed in accordance with the 10-20 international electrode system and two additional electrodes were set at FT9-FT10 and one at electrocardiogram.

The events registered on vEEG were reviewed by a neurologist trained in epileptology, who made the diagnosis of PNESs, and then classified them according to their semiology into H, A, FM or SS based on the classification of Magaudda et al.^2^. In doubtful cases, the vEEG was discussed between two or three neurologists trained in epileptology. Unless final agreement with the diagnosis of PNES was achieved among these experts, the patient was not included.

Seizures and epilepsy was defined in accordance with the latest ILAE definition (2017)^3^.

## RESULTS

**Figure 1.**
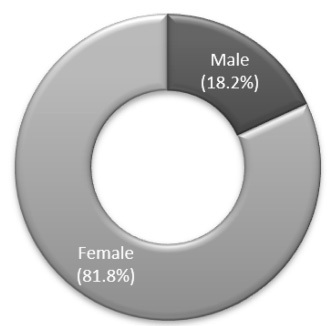
Sex distribution.

**Figure 2.**
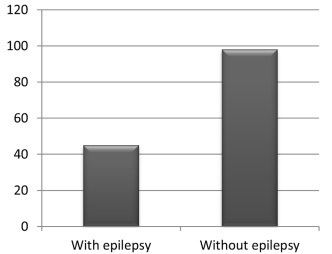
Number of patients with (45) and without (98) associated epilepsy.

**Figure 3.**
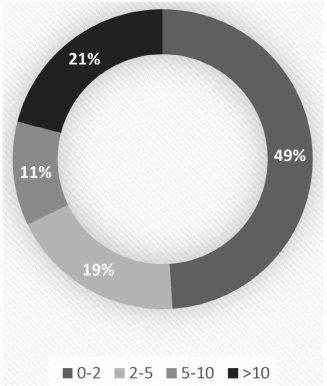
Length of time with epilepsy, since the first clinical event. Data in years.

A total of 376 patients were admitted to the vEEG unit. A total of 143 patients were diagnosed as presenting PNES. Twenty-six of these patients (18.2%) were men and 117 (81.8%) were women (Figure 1). Out of the total number of patients admitted to the vEEG unit, 31% were females and 69% were males. The mean age of the PNES patients was 33.74 years (range: 18-83). Additionally, 31.5% (n = 45) had been diagnosed with epilepsy too (Figure 2). The group of patients with epilepsy included patients who had two types of events seen on the vEEG: clinical events characteristic of PNES in the absence of EEG alterations; and typical epileptic seizures with electroencephalographic correlation. In contrast, the group of patients without epilepsy only presented clinical events characteristic of PNES, without alterations on the vEEG. Most of the nonepileptic patients were women (85.7%). The length of time since the first clinical event was 0 to 2 years in 49% of the cases, 2 to 5 years in 18.8%, 5 to 10 years in 11.2% and over 10 years in 21% (Figure 3). No significant differences were observed between patients with and without epilepsy. Interictal electroencephalographic (EEG) abnormalities were found in 44.1% of the 143 patients. Pathological interictal EEG findings were observed in 80% of the patients who also had epilepsy. Nonepileptic patients showed interictal EEG abnormalities in 27.6% of the cases.

The mean number of antiepileptic drugs (AEDs) administered to each patient was 2.33 (range: 0 to 8). The mean number of AEDs used by the group of nonepileptic patients was 1.73, and by the epileptic group was 3.62 (p < 0.001).

Irrespective of gender, 48.3% of all of the patients had some associated unspecified psychiatric disorder. Also, many patients mentioned during their admission interview that they had a previous history of physical or psychological trauma or stress. Hospitalization due to PNESs was necessary in 22.4% of the cases at some point.

**Figure 4.**
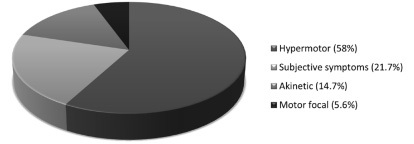
Semiological classification.

In accordance with the semiological classification of Magaudda et al.^2^, the types of PNESs were: hypermotor (H) in 58% of the cases (mainly characterized by tonic or clonic generalized movements; generalized tremors; whole-body rigidity and/or movements; pelvic thrusting; and/or side-to-side head movements); subjective symptoms (SS) in 21.7% (experiential phenomena and paresthesia); akinetic (A) in 14.7% (mainly characterized by unresponsiveness and absence of movement); and focal motor (FM) in 5.6% (focal hand/limb tonic or clonic movement) (Figure 4).

The predominant type in both genders was H, followed by SS in female patients (23.9%) and A in male patients (19.2%). Among the patients without epilepsy, the most frequent manifestation was H in 59.2% of the cases followed by SS in 23.5%, A in 11.2% and FM in 6.1%. Among the patients with epilepsy, H was the most frequent manifestation, in 55.5% of the cases, followed by A in 22.2%, SS in 17.7% and FM in 4.4%. No significative difference was observed between the two groups (Table 1).

**Table 1.** Types of semiological classification with and without epilepsy.

Semiological type	Total	F/M	Without epilepsy	F/M	With epilepsy	F/M
Hypermotor	83	67/16	58 (59.1)	49/9	25 (55.5)	18/7
Akinetic	21	16/5	11 (11.22)	9/2	10 (22.2	7/3
Subjective symptoms	31	28/3	23 (23.4)	22/1	8 (17.7)	6/2
Focal motor	2	6/2	6 (6.1)	4/2	2 (4.4)	2/0
Total	143		98		45	

F: female; M: male.

## DISCUSSION

PNESs tend to occur in patients in the age range from 20 to 30 years, although the overall range of age occurrence also includes children and elderly patients^4^. Since this study only included adults, the mean age was 33.7.

An accurate diagnosis is the starting point for effective management of patients with PNESs. Misdiagnoses, i.e. assuming that events are epileptic seizures, lead to improper treatments and a significant risk of iatrogenic injury and morbidity, with high costs for the patient and the healthcare system^5^. However, making this diagnosis always poses a challenge due to the percentage of patients with PNESs and concomitant epileptic seizures, which reached 31.5% in our series. This finding was similar to what has been reported in the literature^6^.

Furthermore, the presence of interictal EEG abnormalities in up to 22% of the subjects with PNESs according to some series, and in 27.6% of the cases in ours, remains a drawback in the search for an early diagnosis. Adoption of routine EEG as a diagnostic tool used in isolation can also lead to a misdiagnosis of epilepsy. Delayed diagnoses (over 10 years in 21% of the patients) shed light upon the importance of the early use of more specific diagnostic tools in cases of clinical suspicion of PNESs, or repetitive events that do not respond to standard treatments^7^.

vEEG is more accurate than EEG for making a differential diagnosis. It enables analysis on semiology and EEGs during and between the events. A normal wakefulness EEG in association with semiological manifestations compatible with PNESs, or in a patient who remained unconscious with eyes closed, with no external motor manifestations, is highly suggestive of nonepileptic events.

However, vEEG techniques also have some drawbacks. It is very important to differentiate artifacts that are secondary to PNES movements: EEGs in the hypermotor type of PNESs usually show bizarre high amplitude activity due to muscle and movement artifacts, which are usually superimposed on a normal awake EEG^8^. Moreover, special attention is needed in interpreting ocular or eyelid movements, which can generate rhythmic artifacts in bifrontal or anterior temporal electrodes (Fp1-2, F7-8). In some situations, it is necessary to repeat the vEEG study to establish the diagnosis with certainty. Up to half of the patients may not show the habitual episodes spontaneously during monitoring, and some of these cannot even be triggered through induction techniques.

In addition, not all episodes of epileptic nature translate into electroencephalographic paroxysmal activity that can be picked up using surface electrodes. At this point, adequate analysis of the clinical manifestations present in the seizure and thorough knowledge of the different manifestations that can be observed in the different types of epileptic seizures, are highly important^9^. The gold standard includes a combination of vEEG, the patient’s medical history and the reports of witnesses.

Furthermore, not all centers have vEEG and, as mentioned earlier, in some patients the events cannot be registered^7^. Even though home videos are a feasible alternative for observing seizures, their diagnostic sensitivity is lower than that of vEEG. Although some clinical signs provide evidence of PNESs, such as long duration, gradual onset, asynchronous movements that increase and decrease in intensity, pelvic thrusting, closed eyes, side-to-side head shaking and ictal crying, none of these are specific. Since different types of epileptic seizures can be considered to be PNESs, a semiological classification can usefully increase diagnostic certainty.

A recent review of the literature performed by Asadi-Pooya^10^ identified 15 PNES classifications reported in the literature. Three of them (Meierkord et al.^11^, Gröppel et al.^12^ and Seneviratne et al.^13^) were previously applied by Dhiman et al.^8^ to a group of 82 patients, but 40 to 65% of their cohort was unclassifiable, so they proposed a classification system consisting of five items. Four of this five items were non-motor, and 34% of their cohort had mixed patterns. We believe that the Magaudda classification is simpler and better characterizes the different subtypes of PNESs, according to their predominant semiological features^2^. Asadi-Pooya recently modified the Magaudda classification into: motor: generalized or focal; and non-motor: akinetic and subjective symptoms; and also included a mixed semiology category^10^. Although the Asadi-Pooya modified system and the original Magaudda system are similar, the Magaudda classification better stratifies the semiological differences between PNESs, which is the basis for correct identification of these events, taking into account that EEGs are usually misleading or obscured by artifacts during the episode.

During the course of this study, we came to understand that the Magaudda classification is a practical and clinically suitable system. Through this system, more than half of our patients with PNESs had events belonging to class H, which is the group in which the symptoms are most commonly confused with epilepsy, especially in emergency rooms, thereby leading to unnecessary hospitalizations. Hence, presence of this type of motor episode should draw the treating physician’s attention to the possibility of PNESs. Along these lines, De Paola et al.^14^ developed an interesting semiological bedside tool to differentiate these events from epileptic seizures.

In the literature^15^, it is indicated that about 70% of patients with PNESs have other psychogenic disorders. This percentage is higher than what we observed in our series (48.3%). More than 70% of patients report a history of psychological trauma, within which those of a sexual nature account for more than 40%^16^.

The costs of treatment for these patients not only include overuse of AEDs, which reached up to 8 drugs, with a mean of 2.33 drugs per patient in our series, and the subsequent risk of unnecessary adverse effects and increased morbidity. They also include hospitalization of individuals who arrive at emergency rooms with a suspicion of epileptic seizures (22.4% in our series), which gives rise to increased waste of institutional and human resources, which could be avoided. The differences observed between the two groups (epileptic vs nonepileptic patients), with a high number of AEDs in the second group may have been because of the greater frequency and/or number of types of events.

This report has some limitations relating to the retrospective nature of the data collection and application of the Magaudda classification. On the other hand, we made a thorough analysis on the semiological manifestations in conjunction with medical history and EEG interpretation during the events, in order to make the PNES diagnoses. Nonetheless, no patients underwent invasive EEG exploration, and this was entirely a clinical descriptive report.

The proposed semiological classification of PNESs, taking into account the clinical features described, was found to be a relevant tool that general neurologists can use to characterize these events in their daily practice. Correct use of this classification, along with vEEG, in the case of an appropriate clinical suspicion, enabled us to reach accurate and early diagnoses, thus reducing morbidity and, possibly, the high costs associated with PNESs.
